# Patient satisfaction and patient-reported outcomes do not vary by BMI class in total hip arthroplasty

**DOI:** 10.1007/s00590-024-03894-x

**Published:** 2024-03-15

**Authors:** Nickelas Huffman, Ignacio Pasqualini, Roberta E. Redfern, Trevor G. Murray, Matthew E. Deren, Craig L. Israelite, Charles L. Nelson, Dave Van Andel, Jason M. Cholewa, Mike B. Anderson, Alison K. Klika, John P. McLaughlin, Nicolas S. Piuzzi

**Affiliations:** 1 Department of Orthopedic Surgery, Cleveland Clinic Foundation, Orthopedic and Rheumatology Institute, Cleveland, OH 44195 USA; 2 Clinical Affairs, Zimmer Biomet, Warsaw, IN 46580 USA; 3 Department of Orthopaedic Surgery, Penn Medicine, Philadelphia, PA 19104 USA; 4 Department of Biomedical Engineering, Cleveland Clinic Foundation, Cleveland, OH 44195 USA

**Keywords:** Patient reported outcomes, Obesity, Body mass index, Total hip arthroplasty, Satisfaction, Hip disability and Osteoarthritis Outcome Score for Joint Replacement

## Abstract

**Purpose:**

Obesity has been identified as a risk factor for postoperative complications in patients undergoing total hip arthroplasty (THA). This study aimed to investigate patient-reported outcomes, pain, and satisfaction as a function of body mass index (BMI) class in patients undergoing THA.

**Methods:**

1736 patients within a prospective observational study were categorized into BMI classes. Pre- and postoperative Hip disability and Osteoarthritis Outcome Score for Joint Replacement (HOOS JR), satisfaction, and pain scores were compared by BMI class using one-way ANOVA.

**Results:**

Healthy weight patients reported the highest preoperative HOOS JR (56.66 ± 13.35) compared to 45.51 ± 14.45 in Class III subjects. Healthy weight and Class III patients reported the lowest (5.65 ± 2.01) and highest (7.06 ± 1.98, *p* < 0.0001) preoperative pain, respectively. Changes in HOOS JR scores from baseline suggest larger improvements with increasing BMI class, where Class III patients reported an increase of 33.7 ± 15.6 points at 90 days compared to 26.1 ± 17.1 in healthy weight individuals (*p* = 0.002). Fewer healthy weight patients achieved the minimal clinically important difference (87.4%) for HOOS JR compared to Class II (96.5%) and III (94.7%) obesity groups at 90 days postoperatively. Changes in satisfaction and pain scores were largest in the Class III patients. Overall, no functional outcomes varied by BMI class postoperatively.

**Conclusion:**

Patients of higher BMI class reported greater improvements following THA. While risk/benefit shared decision-making remains a personalized requirement of THA, this study highlights that utilization of BMI cutoff may not be warranted based on pain and functional improvement.

## Introduction

The prevalence of obesity is rapidly increasing within the United States [1]. With obesity functioning as an independent risk factor for the development of osteoarthritis (OA), a common indication for total hip arthroplasty (THA) [2], a large proportion of patients requiring arthroplasty are overweight or obese [3, 4]. Furthermore, the overall number of THAs performed is expected to rise exponentially [5]. Although there is evidence demonstrating increased rates of postoperative complications in obese patients undergoing THA [6, 7], studies investigating the impact of patients’ body mass index (BMI) on functional outcomes provide conflicting results. For example, Shevenell et al. [6] reports greater pain and less functional improvement among morbidly obese patients 1 year after THA when compared to healthy weight individuals, whereas Robertson et al. [3] reports no differences in absolute or change in patient reported outcome measures (PROMs) between any weight category for patients undergoing THA. These studies are not alone in their conflicting results [8–11]. Many studies do not analyze the minimal clinically important difference (MCID) when investigating the effects of obesity on postoperative pain and function in THA patients. MCID provides valuable information for healthcare providers as it can identify small changes in outcomes that patients perceive as beneficial [12].

In addition to evaluating patient pain and function, it is also important to obtain information from patients regarding their satisfaction postoperatively, as it is estimated that up to 15% of patients remain dissatisfied following THA [13, 14], with obesity as a possible risk factor for dissatisfaction [15]. This study aimed to investigate patient-reported pain, function, and satisfaction among BMI classes in a large cohort of patients at 90-days and 1-year after THA.

## Methods

**Fig. 1:**
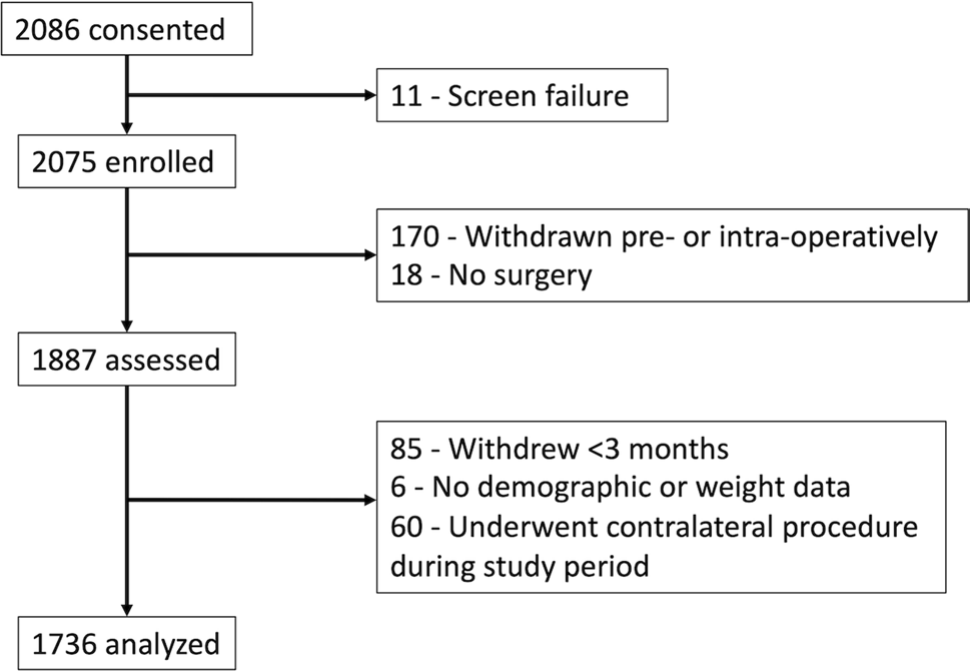
Strobe diagram depicting inclusion and exclusion criteria for the current study

Patients enrolled in a prospective observational study (NCT03737149) investigating the effectiveness of a smartphone-based care management platform (mymobility®, Zimmer Biomet, Warsaw, IN) between November 2018 and July 2022 were included in analysis if they underwent THA, demographic data including height and weight were recorded, and at least 3 months of follow-up data were available. Patients were eligible for enrollment if they were ≥ 18 years old, owned an Apple iPhone® (Apple Inc., Cupertino, CA), were deemed appropriate for self-directed rehabilitation by their surgeon, and did not require the use of more than a single cane or crutch for ambulation preoperatively. Patients were excluded from participation (Fig. 1) if known current alcohol or drug abuse was present, they were participating in other physical therapy or pain management trials, or were undergoing bilateral arthroplasty (simultaneous or staged ≤ 90 days). Patients who underwent a contralateral procedure during the study period were excluded from this analysis, given the unknown impact of the second surgery on recovery of the index procedure. All patients provided written informed consent upon enrollment; central IRB approval was obtained prior to commencement of the study. Patients received pre- and postoperative education, exercises, and delivery of PROMs surveys via the mobile application as previously described [16].

Patients eligible for analysis (n = 1736) were categorized into Centers for Disease Control (CDC) BMI classes by preoperative height and weight. Research staff recorded additional demographics including the following comorbid conditions, which were aggregated to create a continuous variable for comparison: congestive heart failure; coronary artery or valve disease; diabetes; chronic pulmonary disease including asthma, chronic bronchitis, COPD, or emphysema; dementia or Alzheimer’s disease; previous stroke or transient ischemic attack; muscular dystrophy; previous cervical spinal surgery; previous lumbar spinal surgery; history of cancer; chronic kidney disease; liver disease; rheumatoid arthritis; or paralysis. Hip disability and Osteoarthritis Outcome Score for Joint Replacement (HOOS JR) was completed up to 180 days preoperatively, and at 1-, 3-, 6-, and 12-months postoperatively; 89.3% of patients provided PROMs data at 90 days and 65% provided this information at 1 year postoperatively. Joint satisfaction (0–40 points with a higher score indicating greater satisfaction) and numeric rating scale (NRS) pain scores (with a score of 10 indicated worse pain) were collected preoperatively and at 1- and 3-months postoperatively. Survey responses and change from baseline were compared by BMI class using one-way ANOVA with pairwise comparisons at each time interval. Achievement of MCID was determined using previously published distribution-based methods [17] and compared by Chi-squared test. All analyses were performed using SAS Enterprise Guide v7.1 (2014, SAS Institute, Inc. Cary, NC, USA); *p* < 0.05 was considered statistically significant.

## Results

### Demographics

The average age of subjects was 61.9 ± 10.4 years; 890 (51.3%) were female. Patient demographics are presented in Table 1. BMI distribution over the cohort demonstrated 9 (0.5%) underweight, 394 (22.7%) healthy weight, 654 (37.7%) overweight, 378 (21.8%) Class I, 196 (11.3%) Class II, and 105 (6.0%) Class III. On ANOVA, age decreased with increasing BMI class (*p* = 0.0003).

**Table 1:** Demographics

	Underweight (n = 9)	Healthy weight (n = 394)	Overweight (n = 654)	Class I (n = 378)	Class II (n = 196)	Class III (n = 105)	*p* value
Age	62.1 ± 21.8	62.6 ± 10.9	63.1 ± 9.9	61.5 ± 10.5	59.8 ± 9.9	57.7 ± 9.4	**0.0003**
Race–Caucasian	7 (77.8)	329 (83.5)	551 (84.3)	320 (84.7)	165 (84.2)	77 (73.3)	0.12
Preop opioid	0 (0)	31 (7.9)	47 (7.2)	48 (12.7)	21 (10.7)	8 (7.6)	**0.04**
Comorbidities	0.78 ± 0.83	0.78 ± 1.08	0.76 ± 1.13	0.89 ± 1.35	0.97 ± 1.29	1.10 ± 1.26	**0.036**
Preop Dx–OA	9 (100)	383 (97.2)	635 (97.1)	366 (96.8)	190 (96.9)	103 (98.1)	0.98
*Sex*							
Female	7 (77.8)	282 (71.6)	287 (43.9)	160 (42.3)	102 (52.0)	52 (49.5)	** < 0.0001**
Male	2 (22.2)	112 (28.4)	367 (56.1)	218 (57.7)	94 (48.0)	53 (50.5)	
*Approach*							
Anterior	2 (22.2)	170 (43.2)	259 (39.6)	128 (33.9)	73 (37.2)	30 (28.6)	**0.0002**
Direct Lateral	0 (0)	3 (0.8)	7 (1.1)	0 (0)	6 (3.1)	5 (4.8)	
Posterior	7 (77.8)	221 (56.1)	388 (59.3)	250 (66.1)	117 (59.7)	70 (66.7)	

Bold values indicate *p* < 0.05

*Dx* diagnosis, *OA* osteoarthritis

### Pre- and postoperative HOOS JR scores and change in scores

Preoperative HOOS JR decreased with increasing obesity class over the cohort (Table 2A, p < 0.0001). Pairwise comparisons revealed healthy weight patients reported significantly higher preoperative HOOS JR scores than overweight patients, as well as patients in all classes of obesity (Table 2A, all pairwise comparisons *p* < 0.01). Overall, functional outcomes did not vary by BMI class at 1 year postoperatively. Changes in HOOS JR scores from baseline suggest larger improvements with increasing BMI class, with Class III patients reporting an increase of 33.67 ± 15.58 points at 90 days compared to 26.12 ± 17.11 in healthy weight individuals (Table 2A, p < 0.0001). On pairwise comparisons, Class II and III patients reported larger HOOS JR improvements than healthy and overweight patients at 90 days postoperative. Class III patients continued to demonstrate the largest improvement in HOOS JR at 1-year postoperative (41.08 ± 17.07), where healthy weight individuals improved by 34.01 ± 15.23 (*p* = 0.01). Class II patients also experienced significantly greater improvements in HOOS JR at 1-year postoperative (40.10 ± 13.63) compared to healthy weight individuals (*p* = 0.003).

**Table 2:** HOOS JR, pain, and satisfaction absolute scores and change from baseline

	Underweight	Healthy weight	Overweight	Class I	Class II	Class III	*p* value
*A) HOOS JR scores and change from baseline*							
Preop	54.46 ± 12.21	56.66 ± 13.35	53.95 ± 12.14	51.21 ± 12.72	49.66 ± 11.41	45.51 ± 14.45	< 0.0001
30 d	77.03 ± 15.64	73.69 ± 12.39	74.11 ± 12.16	72.29 ± 12.16	72.77 ± 12.80	72.70 ± 12.16	0.23
90 d	80.84 ± 9.54	82.63 ± 14.15	82.52 ± 12.82	80.80 ± 12.60	81.66 ± 12.32	80.37 ± 13.74	0.29
6 m	81.39 ± 13.48	87.43 ± 14.08	86.74 ± 12.76	84.72 ± 12.84	84.97 ± 13.31	85.21 ± 12.64	0.05
1 y	93.22 ± 8.51	90.72 ± 11.71	90.58 ± 12.08	88.51 ± 12.62	89.48 ± 11.82	86.79 ± 13.72	0.06
Δ30 d	22.57 ± 18.84	16.89 ± 15.58	20.02 ± 14.58	21.04 ± 14.87	23.14 ± 15.36	26.25 ± 15.14	< 0.0001
Δ 90 d	26.38 ± 17.20	26.12 ± 17.11	28.47 ± 15.31	29.38 ± 15.89	32.08 ± 15.17	33.67 ± 15.58	< 0.0001
Δ 6 m	26.94 ± 21.10	30.81 ± 16.56	32.47 ± 15.71	32.98 ± 15.22	35.24 ± 14.84	38.45 ± 14.45	0.0007
Δ 1 y	38.19 ± 16.25	34.01 ± 15.23	36.70 ± 15.75	36.34 ± 15.94	40.10 ± 13.63	41.08 ± 17.07	0.002
*B) Pain scores and change from baseline*							
Preop	6.22 ± 1.30	5.65 ± 2.01	5.80 ± 1.99	6.32 ± 2.07	6.36 ± 1.71	7.06 ± 1.98	< 0.0001
30 d	2.38 ± 1.51	2.53 ± 1.97	2.57 ± 2.00	2.65 ± 2.02	2.64 ± 2.16	2.42 ± 1.97	0.93
90 d	3.38 ± 2.33	1.63 ± 1.90	1.62 ± 1.99	1.64 ± 1.89	1.24 ± 1.69	1.85 ± 2.28	0.02
Δ 30 d	- 3.88 ± 1.96	- 3.11 ± 2.56	- 3.20 ± 2.50	- 3.64 ± 2.50	- 3.69 ± 2.60	- 4.35 ± 2.52	0.0002
Δ 90 d	- 2.88 ± 2.95	- 3.98 ± 2.62	- 4.15 ± 2.54	- 4.64 ± 2.66	- 5.01 ± 2.12	- 5.04 ± 2.99	< 0.0001
*C) Satisfaction and change from baseline*							
Preop	10.67 ± 3.46	12.18 ± 7.59	11.86 ± 7.21	10.6 ± 7.14	10.25 ± 6.72	9.24 ± 6.81	< 0.0001
30 d	31.56 ± 6.39	25.11 ± 13.0	25.10 ± 13.29	25.56 ± 12.98	25.5 ± 13.04	27.35 ± 12.48	0.42
90 d	28.25 ± 5.90	31.25 ± 9.96	31.20 ± 10.42	31.66 ± 10.08	32.57 ± 9.68	31.12 ± 11.29	0.65
Δ 30 d	20.89 ± 7.88	13.68 ± 14.12	13.96 ± 14.28	15.56 ± 14.0	16.20 ± 13.83	18.33 ± 13.69	0.008
Δ 90 d	17.75 ± 7.29	18.98 ± 12.19	19.28 ± 12.45	21.03 ± 12.12	22.39 ± 11.09	21.30 ± 12.99	0.02

*HOOS JR* Hip disability and Osteoarthritis Outcome score for Joint Replacement

### Achievement of MCID for HOOS JR

The Class II obesity group demonstrated the largest proportion reaching MCID for HOOS JR at 90 days postoperative (96.5%) compared to healthy weight individuals (87.4%) (Table 3; *p* = 0.003). The distribution of patients reaching MCID for HOOS JR at one year did not differ by class (94.6%-100%, *p* = 0.35).

**Table 3:** Achievement of MCID for HOOS JR

	Underweight	Healthy weight	Overweight	Class I	Class II	Class III	*p* value
30 d	7/9 (77.8)	267/377 (70.8)	503/617 (81.5)	305/362 (84.3)	155/183 (84.7)	92/100 (92.0)	< 0.0001
90 d	7/9 (77.8)	311/356 (87.4)	539/582 (92.6)	310/337 (92.0)	167/173 (96.5)	89 (94.7)	0.003
6 m	8/9 (88.9)	297/325 (91.4)	497/525 (94.7)	302/316 (95.6)	163/166 (98.2)	82/84 (97.6)	0.02
1 y	8/8 (100)	395/410 (96.3)	245/259 (94.6)	247/257 (96.1)	129/130 (99.2)	62/65 (95.4)	0.35

*MCID* minimal clinically important difference, *HOOS JR* Hip disability and Osteoarthritis Outcome score for Joint Replacement

### Pre- and postoperative pain and satisfaction scores and change in scores

Preoperative patient-reported pain scores increased with BMI class (Table 2B; *p* < 0.0001). Class I–III patients reported higher preoperative pain than those with healthy weights preoperatively (all, *p* < 0.001). Similarly, Class I–III individuals reported greater preoperative pain than those in the overweight class (all, *p* < 0.01). Change in pain score at 90 days postoperatively varied by class (Table 2B; *p* < 0.0001), with the largest change in the Class III obesity group − 5.04 ± 2.99 compared to − 3.98 ± 2.62 pain reduction in healthy weight patients *(p* = 0.01). When compared to healthy weight individuals, Class I-III obesity groups exhibited significantly greater change in pain at 90 days postoperatively on pairwise comparisons (all pairwise comparisons, *p* < 0.05). Change in satisfaction also varied across BMI classes (Table 2C; *p* = 0.02) with the largest change in the Class II obesity group at 90 days postoperatively 22.39 ± 11.09, compared to 18.98 ± 12.12 points improvement in healthy weight individuals (*p* = 0.04).

## Discussion

The current results demonstrate worse preoperative HOOS JR and pain scores with increasing BMI. However, postoperatively, there was no identified difference between absolute HOOS JR scores between the different BMI classes. Greater improvements were observed in HOOS JR scores at all postoperative time points, pain scores at 90 days postoperative, and satisfaction scores at 90 days postoperative for patients with greater BMI compared to individuals in the healthy weight class. Furthermore, a significantly greater proportion of patients with Class II obesity achieved MCID for HOOS JR at 90 days postoperatively compared to healthy weight individuals, but there was no difference in the achievement of MCID for HOOS JR between BMI groups at 1 year postoperatively. These results contribute to elucidating the role BMI plays in postoperative PROMs after THA.

Previous studies have determined that patients with a greater BMI often have worse preoperative PROMs [6, 18], and these results align well with the current study. Beyond these findings, however, there are many conflicting results on the effect BMI has on postoperative THA PROMs. Similar to the current study, prior studies have demonstrated excellent functional outcomes, with 1 year postoperative THA patients experiencing greater improvement in pain and function, as measured by the HOOS Pain and HOOS Physical function Shortform (PS), respectively, as BMI increased [18]. Sato et al. [19] analyzed postoperative HOOS JR scores and reported that obese patients recovered at a significantly faster rate compared to non-obese patients up until 3 months postoperatively (*p* < 0.05). However, the differences in 1-year postoperative HOOS JR scores between BMI groups were similar to the differences reported at preoperative baseline evaluation [19]. Other studies have also observed no difference in outcomes between obesity classes postoperatively, including a study conducted by Mukka et al. [20] that found at 1-year follow-up, all BMI classes demonstrated statistically significant and clinically relevant improvements in all Health-Related Quality of Life measures compared to preoperative assessment (*p* < 0.05). Finally, studies also report that patients with greater BMI have worse pain and function outcomes after THA. Shevenell et al. [6] reported that patients with Class III obesity were found to have greater pain (*p* = 0.041) and worse functional improvement (measured by HOOS JR, *p* = 0.002) 1 year after THA compared to healthy weight individuals. Our results demonstrate greater subjective functional improvements and pain reduction in higher obesity classes, which contrast with this previous report.

Several studies report similar satisfaction rates among obese and non-obese patients after THA [4, 11, 21]. One study specifically found that at 1-year following THA, there was no significant difference in satisfaction among different BMI classifications [22]. The results of the current study align well with previous results. Although our study did not collect satisfaction data at 1 year postoperatively, our results show that satisfaction rates were similar among all BMI classes at both 30 and 90 days postoperatively, suggesting no relationship between BMI and postoperative THA satisfaction.

Data on MCID after THA based on obesity categories is scarce. Among different BMI groupings, studies have found that similar proportions within each BMI group have achieved MCID for OHS, WOMAC, and SF-36 PCS [21]. These results are consistent with our findings. However, other studies report that at 1-year postoperative THA, increasing BMI was a significant risk factor for failing to achieve MCID for HOOS PS [10]. With regards to the current finding of Class II demonstrating the greatest proportion of patients achieving MCID for HOOS JR at 90 days (96.5%), it is important to note that Class III demonstrated a similar proportion achieving MCID at 90 days (94.7%). This minimal difference in the proportion achieving MCID at 90 days may not be clinically relevant. However, there may be a difference between BMI groups because the HOOS JR considers a patient’s functionality, and it is possible that patients’ increasing weight may begin to restrict their functionality postoperatively, regardless of THA outcomes. Future studies should aim to elucidate potential reasons for these findings.

There are several reasons the pre- and postoperative PROM results may differ between studies. First, there are many validated PROM questionnaires commonly used in orthopaedics. Although questionnaires often fall into categories and assess patient pain, function, or satisfaction, each questionnaire may differ in results, making it difficult to compare studies utilizing different PROMs. Furthermore, many studies examine PROMs at various postoperative intervals, and the different methods can again lead to different results. Also, the BMI classifications that studies use to compare PROMs differ within the literature, with some studies comparing only morbidly obese patients (BMI ≥ 40) to healthy weight patients [6] or only obese patient (BMI ≥ 35) to healthy weight patients [23], while others use the World Health Organization BMI classifications [3]. Finally, while many studies report statistically significant differences in PROMs between BMI classes, it is important to recognize that some statistically significant findings are not clinically significant. For example, Judge et al. [8] found that patients with Class II obesity had an OHS that was, on average, 2.34 points lower than healthy weight individuals 1 year after THA. Although statistically significant, the authors concluded that this result was not clinically meaningful, as this same Class II obesity group experienced a 22.2-point improvement in OHS from pre- to post-operation [8]. At each interval in our study, the difference in improvements between healthy weight and Class III was greater than previously established MCID values [17].

### Limitations

The current study did not evaluate operative and postoperative complication rates among patients. We acknowledge the need to consider surgical risks as an important factor in preoperative evaluation. Reports in the literature provide more consistent results suggesting increased postoperative complications such as infection, readmission, and reoperation in obese patients [6, 7, 21]. These complications, however, are outside the scope of the current study as our objective was to compare pain, function, and satisfaction as a function of BMI classification. Interestingly, one study reported the paradoxical relationship of increased readmission rates for patients who decreased their BMI prior to THA [24]. Our classification of patients into obesity categories was based on their preoperative weights at study enrollment, we did not collect weight information on the date of surgery. It is possible that some patients may have reduced their BMI prior to surgical intervention, though it is unlikely this would have affected BMI categorization. Furthermore, the current study did not record preoperative OA severity. Preoperative OA severity may impact postoperative outcomes, and thus is an important factor to consider when analyzing postoperative outcomes. A recent systematic review conducted by Beiene et al. [25] found that roughly 85% of studies did not include a specific level of preoperative OA severity as an inclusion criterion [25]. It is important for future studies to begin including preoperative OA severity because without this information there can be variability among cohorts resulting in decreased external validity of study results. Finally, the current study investigated patient satisfaction and pain scores only up until 90 days postoperatively; it may be important for future studies to analyze these outcomes at later postoperative time periods.

## Conclusion

Overall, patients of higher BMI class reported greater improvements in hip function, pain, and satisfaction, as demonstrated by their changes in scores, shortly after THA compared to healthy weight individuals. Individuals with the highest BMI (Class III obesity) appeared to appreciate the greatest benefits as measured by changes in HOOS JR, satisfaction, and pain scores. However, there was no difference in absolute satisfaction scores at 3 months, absolute HOOS JR scores at 1 year, and achievement of MCID for HOOS JR at 1 year, providing evidence of the promising outcomes following THA regardless of BMI class. While risk/benefit shared decision making remains a personalized and case-by-case requirement of THA, this study highlights that the utilization of BMI cutoff points alone may not be warranted based on pain and functional improvement.
